# Effects of Communication Strategies on Treatment Adherence and Success in Tuberculosis: A Systematic Review and Meta‐Analysis

**DOI:** 10.1111/tmi.70013

**Published:** 2025-08-13

**Authors:** Omar Oliveira Meira, Luiz Gustavo Ribeiro Silva, Raquel Fonseca Sales, Renata Maria Colodette, Lucas Borges Gomes Ferreira Pinto, Emily de Souza Ferreira, Rosângela Minardi Mitre Cotta, Tiago Ricardo Moreira

**Affiliations:** ^1^ Graduate Program in Health Sciences, Department of Medicine and Nursing Federal University of Viçosa Viçosa Minas Gerais Brazil; ^2^ Department of Medicine and Nursing Federal University of Viçosa Viçosa Minas Gerais Brazil; ^3^ Dentistry Course Health Sciences Center, Viçosa University Center (UNIVIÇOSA) Viçosa Minas Gerais Brazil; ^4^ Graduate Program in Nutrition Science, Department of Health Nutrition Federal University of Viçosa Viçosa Minas Gerais Brazil

**Keywords:** communication, meta‐analysis, treatment adherence and compliance, tuberculosis

## Abstract

**Introduction:**

Tuberculosis, although curable, presents challenges related to treatment adherence, which compromises treatment effectiveness. Individual, social and structural barriers interfere with patients' ability to properly follow the therapeutic regimen, thereby impacting treatment outcomes. Given the limitations of the conventional healthcare model, which relies primarily on in‐person consultations and standard treatment protocols without additional adherence support technologies, new approaches have been explored to improve patient outcomes. This study seeks to identify effective communication approaches in this context.

**Objective:**

To identify the most effective communication strategies to optimise treatment adherence and improve therapeutic success in patients diagnosed with tuberculosis.

**Methods:**

A systematic review with meta‐analysis was conducted. We included studies available in the MEDLINE (via PubMed), EMBASE and SCOPUS databases, with publication dates between January 2005 and December 2024. The primary outcomes were adherence to and success in tuberculosis treatment.

**Results:**

This systematic review included 17 studies on tuberculosis treatment adherence. Of these, 12 were included in the meta‐analysis for adherence and 8 for treatment success. The most effective strategies for adherence were community education (2 studies; RR: 0.25, 95% CI: 0.11–0.56) and video observed therapy (VDOT) (2 studies; RR: 0.29, 95% CI: 0.21–0.40). The combination of electronic devices with SMS also showed positive results (3 studies; RR: 0.53, 95% CI: 0.37–0.77). SMS alone (5 studies) and electronic devices alone (3 studies) were not effective. For treatment success, only the combination of electronic devices with SMS (RR: 0.31, 95% CI: 0.17–0.55) and community education (RR: 0.51, 95% CI: 0.40–0.64) were effective.

**Conclusion:**

The combination of electronic devices with SMS and community education is an effective strategy for improving adherence and therapeutic success in tuberculosis treatment. Isolated interventions with SMS or electronic technologies did not show significant results. Adapting approaches to local realities is crucial for optimising outcomes.

## 1 Introduction

According to the 2024 Global Tuberculosis Report from the World Health Organisation (WHO), it is estimated that more than 10 million people fall ill with tuberculosis each year worldwide [1]. The number of people with tuberculosis in 2023 was the highest ever recorded since global monitoring of the disease began in 1995 [2]. Currently, tuberculosis has once again become the leading cause of death globally from a single infectious agent, with 80% of cases and deaths occurring in low‐ and middle‐income countries [3].

The WHO has identified three global lists of countries with a high tuberculosis burden for the period 2021 to 2025 [4]. These lists categorise countries based on their overall high tuberculosis burden, high burden of TB/HIV coinfection and high burden of drug‐resistant tuberculosis.

Tuberculosis is a serious but curable disease. If the treatment regimen is appropriate and the correct doses of medications are taken regularly for a sufficient duration, nearly 100% of patients can be cured [5]. However, the approach to tuberculosis treatment remains challenging, as it must take into account both individual and collective health contexts. In this regard, despite the high efficacy of the anti‐tuberculosis regimen, treatment effectiveness—that is, the proportion of patients who are actually cured by the end of the regimen—varies considerably. The global treatment success rate for patients treated with first‐line regimens is approximately 86%, but it varies by region, ranging from 74% in the Americas to 91% in the Eastern Mediterranean [6].

Currently, the treatment of this disease in adults and adolescents consists of a basic regimen with a total duration ranging from 6 to 12 months. The first 2 months comprise the intensive phase, followed by the maintenance phase in the subsequent months. In both phases, the patient must take tablets containing different drugs in fixed‐dose combinations, in a single daily dose, preferably on an empty stomach [7], with the number of tablets indicated varying according to the patient's weight [8].

Poor adherence to treatment contributes to the continuation of the disease transmission cycle and exposes the individual to active infection for longer periods, increasing the risk of relapse and death. The emergence of drug‐resistant tuberculosis is also a concern in this context [9]. Thus, adherence to drug therapy is a critical factor in determining therapeutic success.

Patients' difficulty in completing treatment can be understood as the result of a combination of independent factors. Structural factors include poverty, high financial costs, gender discrimination, legal aspects and communication approaches that may create barriers between patients and health services. Personal factors involve knowledge level, beliefs and attitudes toward treatment, as well as the way individuals interpret illness and health. Factors related to health services include the organisation of care, monitoring of disease progression, the communication strategies adopted by health professionals and side effects of medications. Lastly, the social context—such as stigma and the level of support from family, the community and the household environment—also greatly influences treatment adherence [10]. Therefore, without proper support, a significant number of tuberculosis patients may discontinue treatment before completion or follow it irregularly [9].

Given the importance of drug therapy for both the patient and public health, approaches to ensure adherence to pharmacological treatment are a key aspect of therapeutic management. Among these approaches, Directly Observed Therapy (DOT) involves patients taking their daily medication doses under the direct observation of a healthcare professional. This strategy has been used as a standard practice in various tuberculosis treatment programmes [11].

However, the implementation of DOT faces difficulties due to the human resource requirements for long‐term encounters, and for people with tuberculosis, directly observed treatment demands considerable time, potentially interfering significantly with employment, family obligations and other daily activities. It may also be perceived as paternalistic and intrusive. Thus, its implementation tends to be challenging and, in most contexts, unfeasible, representing an economic and social burden for both tuberculosis patients and health programmes in low‐income countries [12].

Recently, WHO has recommended that countries maximise the use of digital adherence technologies (DATs). These technologies can improve tuberculosis treatment outcomes and reduce costs [13]. With the increasing use of mobile phones worldwide, these tools offer an opportunity to facilitate direct communication between healthcare professionals and people with the disease [14].

Among the DATs approved by WHO are: short message service/text messaging (SMS), medication event reminder monitoring systems (MERM) and video‐supported directly observed therapy (VDOT) [13].

Considering these technologies and the previously mentioned challenges related to tuberculosis treatment adherence, there is a need to investigate which communication strategies are effective in this context. Therefore, the objective of this study was to analyse which communicative approaches contribute to improving therapeutic adherence and treatment success among people with tuberculosis.

## 2 Methods

### 2.1 Eligibility Criteria

The research question was developed based on the PICOS strategy, as follows: P (participants): patients with tuberculosis; I (interventions): communication strategies; C (comparisons): patients receiving standard treatment; O (outcomes): treatment adherence and success; and S (study design): cross‐sectional studies, cohorts, clinical trials and quasi‐experimental studies.

Original studies conducted from January 2005 onward were included, with no restrictions regarding study location, but limited to articles published in English or Portuguese. The selected period is justified by the fact that the component “communication” was incorporated into the directly observed treatment strategy starting in 2005 [15].

For inclusion in the meta‐analysis, studies were selected that compared communication strategies with usual care and presented pre‐ and post‐intervention data in order to estimate the effectiveness of communication strategies in the target population.

Studies that were not original or from which it was not possible to extract relevant data for analysis—such as letters, editorials, conference proceedings, commentaries, reports, study protocols, pilot studies, abstracts and reviews—were excluded. In addition, articles that did not provide a detailed description of the communication strategies used were also excluded.

### 2.2 Information Sources

The search for articles was conducted in the following databases: Medical Literature Analysis and Retrieval System Online (MEDLINE/PubMed), Excerpta Medica dataBASE (EMBASE) and Scopus. No search was conducted in grey literature. The search was carried out between November 18, 2024, which marks the date of the last search.

### 2.3 Search Strategy

The search strategy was based on the analysis of keywords used in the literature and in previous studies. Using the identified keywords, appropriate descriptors were selected for the search from DeCS/Mesh (Health Sciences Descriptors) and Emtree.

The descriptors and Boolean operators used to perform the search across all databases were: (Tuberculose OR Tuberculosis) AND (Comunicação OR Communication OR “communication strategies” OR “Estratégias de Comunicação”) AND (“Cooperação e Adesão ao Tratamento” OR “Treatment Adherence and Compliance” OR “patient compliance”) AND (Pacientes OR Patients).

### 2.4 Selection Process

The studies identified in the aforementioned databases were managed using the StArt program (State of the Art through Systematic Review).

The study selection was carried out independently by two researchers (O.O.M. and Y.L.G.). Initially, duplicate studies extracted from different databases were excluded. Then, potentially eligible articles were identified (according to the inclusion and exclusion criteria) through the screening of titles and abstracts.

Subsequently, the studies selected in the previous stage were read in full. Discrepancies in the selection between the two researchers were discussed, and a third researcher assisted in the final decision regarding the selection of studies included in this systematic review.

### 2.5 Data Collection Process

A standardised file was used to extract useful data for the systematic review. Two authors (O.O.M. and Y.L.G.) performed the data extraction and accuracy analysis. The data extracted from all selected studies were managed using Microsoft Excel.

### 2.6 Data Items

The following data were extracted: title, authors, year of publication, year of the study, country where the study was conducted, type/design of the study, number of study participants, duration of the intervention, primary and secondary outcomes, as well as their definitions according to the classification provided in each study.

During this process, when data were unclear or missing from the original reports, the authors contacted the study investigators directly to obtain clarifications and additional information.

### 2.7 Study Risk of Bias Assessment

The risk of bias analysis of the selected studies was carried out by two reviewers (L.G.R.S. and R.F.S.) using the Newcastle‐Ottawa Scale (NOS) [16] assessment tool for observational (cross‐sectional and cohort) and quasi‐experimental studies. For randomised clinical trials, cluster randomised trials and group randomised trials, the Review Manager 5.4 (RevMan) software [17] was used, applying the RoB 2 (Risk of Bias 2) tool [18]. The risk of bias in the studies was not defined as an exclusion criterion. The synthesis of the risk of bias assessment for all included studies can be seen in Figures 2 and 3 in the Results section of this study.

### 2.8 Effect Measures

The results were synthesised through two meta‐analyses. In the first, the number of individuals who adhered to treatment was considered, while in the second, treatment success was evaluated by comparing the control and intervention groups. The criteria for adherence and success were defined based on the parameters established by the included studies.

To present the results, relative risk was used as the measure of association. The statistical significance of the overall effect of communication strategies was determined using a 95% confidence interval (CI) and a significance level of 5%. All analyses were performed using Stata software, version 11.

### 2.9 Synthesis Methods

The meta‐analyses were performed using a random effects model in Stata software (version 11.0). Heterogeneity was assessed using the chi‐square test (*χ* ^2^) with a 90% significance level (*p* value < 0.10), and its magnitude was determined using the I‐squared (*I* ^2^) statistic [19]. Accordingly, heterogeneity was classified as low, moderate, or high when *I* ^2^‐values were above 25%, 50% and 75%, respectively.

Additionally, the dispersion of individual results in the forest plot was also used to visually assess the presence of statistical heterogeneity. Analyses were conducted using the “metan” command and meta‐regressions (command “metareg”) with the aim of identifying sources of heterogeneity, applying the Knapp and Hartung test [19]. Initially, a univariate analysis was conducted. All variables associated with the outcomes in this analysis (*p* value < 0.20) were included in the final multivariate model. For these analyses, a significance level of 5% was established.

### 2.10 Reporting Bias Assessment

The presence of small‐study effects was also assessed through visual inspection of the funnel plot and the Egger test [20].

### 2.11 Certainty Assessment

The methodological quality of the studies was assessed using critical appraisal tools from the Joanna Briggs Institute [21]. The most appropriate tool was selected for each type of study included in the systematic review. For quasi‐experimental studies, the tool comprised 9 questions; for randomised clinical trials, 13 questions; and for cross‐sectional and cohort studies, 8 questions. Two independent reviewers (O.O.M. and Y.L.G.) conducted the quality assessment and risk of bias evaluation. Discrepancies were resolved through consensus.

Each study received one point for each parameter met in the Joanna Briggs Institute tools. Quasi‐experimental studies were considered of high quality with scores between 7 and 9, moderate quality between 4 and 6 and low quality between 0 and 3. For randomised clinical trials, studies were considered high quality with scores between 10 and 13, moderate quality between 5 and 9 and low quality between 0 and 4. For cross‐sectional and cohort observational studies, high quality was considered with scores between 6 and 8, moderate quality between 4 and 5 and low quality between 0 and 3 points [13]. Quality was not established as an exclusion criterion.

### 2.12 Registration Protocol

The protocol is registered in the International Prospective Register of Systematic Reviews (PROSPERO) under the registration number CRD42023440403. The study was conducted according to the recommendations of the Preferred Reporting Items for Systematic Reviews and Meta‐Analyses (PRISMA) [22].

## 3 Results

### 3.1 Study Selection

**FIGURE 1:**
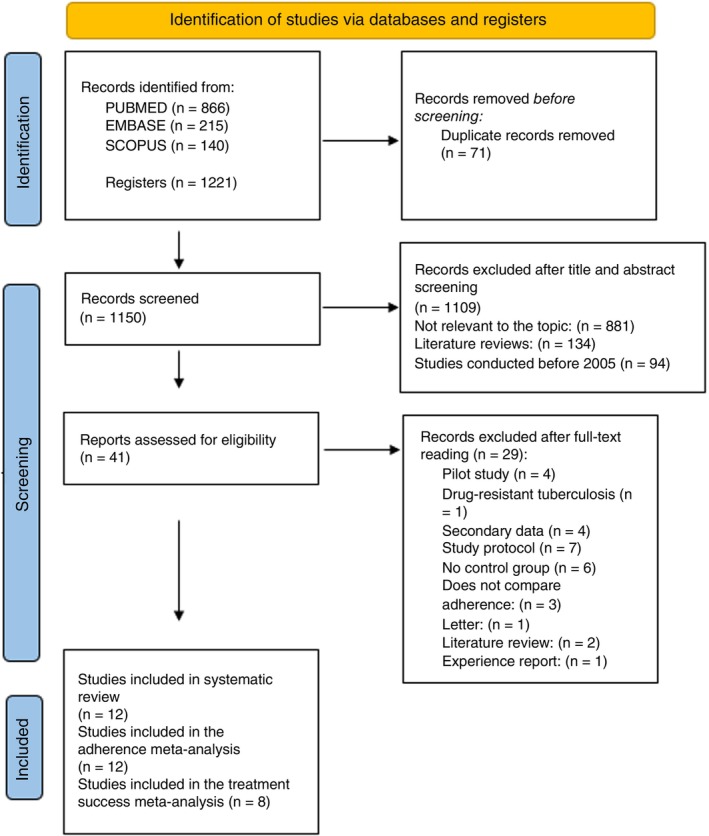
Flowchart of study selection using the PRISMA method.

Figure 1 illustrates the process of article search and selection. A total of 1221 studies were initially identified, of which 1150 remained after duplicates were removed. After screening the titles and abstracts, 41 studies were considered eligible. Following a full‐text review, 12 studies met the eligibility criteria and were included in this systematic review. No additional studies were included after reviewing the references of the accepted articles. All 12 studies selected for the systematic review were also included in the meta‐analysis on treatment adherence. For the meta‐analysis on treatment success, 8 studies were included.

### 3.2 Study Characteristics

The studies were published between 2007 and 2022. Methodological designs included randomised clinical trials, quasi‐experimental studies, cohort studies and cross‐sectional studies, totalling a sample of 4854 individuals. The largest study, conducted in China, included 2197 participants, while the smallest, from the United States, included 61 participants. Data collection was conducted in the United States, Indonesia, Senegal, Moldova, Pakistan, Morocco, Cameroon and Peru.

Regarding methodological quality, cohort, quasi‐experimental and cross‐sectional studies were classified as high quality. Among the randomised clinical trials, four studies were identified as high quality and three as moderate quality [23, 24, 25]. This moderate classification was mainly due to a lack of clarity regarding essential aspects of the study design, particularly concerning allocation concealment and the blinding of participants, healthcare providers and outcome assessors. It is important to emphasise that in behavioural interventions, such as those based on SMS reminders or the use of automated teller machines (ATMs) for reminders, blinding of participants is inherently unfeasible. However, the absence of detailed information on how allocation concealment and blinding of outcome assessors were conducted—or whether these procedures were implemented at all—negatively impacted the methodological quality scores of these studies. Table 1 presents the studies included in this review, indicating the methodological design, type of intervention and the outcomes assessed.

**TABLE 1:** Studies included in the systematic review on communication strategies implemented to improve adherence to pulmonary tuberculosis treatment.

Author, year	Location	Sample	Type of study	Type and duration of intervention	Type and duration of control	Outcomes	Outcome definition	Study quality
Acosta et al. (2022) [23]	Peru	Control: 53 Intervention: 49	RCT	Medication monitoring and reminder system with electronic packaging, web server and SMS sending. If the device was not opened at the scheduled time, up to 3 text messages were sent to the participant; the last one was also sent to a family member and the treatment supervisor, who would then make contact. **Classification**: Electronic devices + SMS **Duration:** 4 months.	DOT in primary healthcare centres administered by a nurse from the TB program, according to national guidelines. **Duration**: 4 months	**Primary**: Treatment success, including patients cured or with treatment completed. **Secondary**: Treatment adherence.	Treatment success was defined as a final diagnosis of “cured” or “treatment completed.” Adherence was assessed by the proportion of missed doses, patients who missed at least one dose and those who missed more than 10% of scheduled doses.	9/13
Herawati (2021) [26]	Indonésia	Control: 62 Intervention: 60	QES	The intervention group used an educational system with videos in Javanese (Ngoko) and the CBIA approach after the first‐day clinic visit, addressing tuberculosis, treatment, reminders and non‐pharmacological aspects. **Classification**: Community education **Duration**: 30 days	Standard care and education on medication administration by healthcare professionals. **Duration**: 30 days	**Primary**: Treatment adherence in control and intervention groups assessed by pill count on days 1 and 30, calculated as the proportion between consumed and prescribed medications.	Respondents were considered to have high adherence if pill count was ≥ 95%, and low adherence if < 95%.	9/9
Ravenscroft et al. (2020) [27]	Moldávia	Control: 90 Intervention: 85	RCT	Treatment used DOT in clinic with digital attendance registration and VDOT, in which the patient recorded a video ingesting the medication. **Classification**: VDOT **Duration**: 22 months (intervention: 4 months)	DOT was conducted at the TB clinic from Monday to Friday. When absence was foreseen, patients received extra doses to take at home. **Duration**: 22 months (intervention: 4 months)	**Primary**: Medication adherence. **Secondary**: Treatment success.	Adherence was assessed by the number of days without medication in 2‐week periods, totalling about eight periods per patient. Treatment success was measured by sputum smear and radiography.	10/13
Browne et al. (2019) [24]	United States	Control: 20 Intervention: 41	RCT	The WOT system, used during the treatment maintenance phase, included an ingestible sensor, a patch on the torso and a paired mobile device. The sensor was **activated** upon ingestion with the medication and sent data to the patch, which then transmitted it to the device. If ingestion was not confirmed on working days, the patient received a text message the same day and a phone call within 24 h. **Classification**: VDOT **Duration**: 4 month	DOT conducted by healthcare professionals. **Duration**: 4 months	**Primary**: Positive detection accuracy of the wireless observed therapy system. **Secondary**: Treatment adherence.	The primary outcome, for each participant and each day, was a binary response on whether the dose was confirmed, either wirelessly (WOT group) or directly by a healthcare worker (DOT group). All observation days were included.	9/13
Park et al. (2019) [28]	Marrocos	Control: 141 Intervention: 206	Cohort	The medication storage device sent real‐time information and reminders about medication intake. In the intervention group, MEMS use began after 1 to 2 weeks of direct supervision at the treatment site, depending on the patient's situation. **Classification**: Electronic Devices + SMS **Duration**: 6 months	DOT during the first two weeks of treatment, followed by self‐administered treatment. **Duration**: 6 months	**Primary**: Treatment adherence. **Secondary**: Treatment success.	**Adherence is defined as the proportion of days a patient correctly took their medication, objectively measured by the Medication Event Monitoring System (MEMS)**. A cured patient was defined as someone with a negative smear at the end of treatment and on one prior occasion. Completed treatment refers to a patient who finished treatment with no evidence of failure but without negative smear records. Treatment success = cured + completed treatment cases.	8/8
Bediang et al. (2018) [29]	Camarões	Control: 142 Intervention: 137	RCT	SMS messages were sent daily to remind, encourage or motivate participants to take their medication. **Classification**: SMS **Duration**: 6 months	DOT conducted by healthcare professionals. **Duration**: 6 months.	Primary: Cure rate at the end of treatment and success. **Secondary**: Self‐evaluation of patients' adherence, consultation attendance, punctuality and satisfaction at 6 months.	Cure was defined as having a negative smear result at the end of treatment. Treatment success included cured patients and those with a negative smear after 6 months. Adherence was measured via a score from 0 to 100% based on self‐assessment at the last three consultations.	12/13
Mohammed et al. (2016) [30]	Paquistão	Control: 1093 Intervention: 1104	RCT	Bidirectional reminders were used to encourage patient engagement. Messages in Urdu (with an English script) included a daily motivational SMS followed by a reminder to reply via SMS or, starting in September 2011, via a missed call to confirm medication ingestion. **Classification**: SMS **Duration**: 6 months	Standard care provided by the patient's local clinic. **Duration**: 6 months	**Primary**: Treatment success. Secondary: Adherence.	Treatment success was defined as patients whose sputum or culture was positive at treatment start but became negative in the last month and at least once before. Treatment completion was defined as patients who completed treatment without a negative sputum or culture result in the final month and one prior occasion. Adherence was self‐reported by each patient.	13/13
Kumboyono (2016) [31]	Indonésia	Control: 45 Intervention: 45	OBS‐CS	Text messages in SMS format. **Classification**: SMS **Duration**: 2 months	DOT provided by healthcare professionals. **Duration**: 2 months	**Primary**: Adherence. **Secondary**: Difference in adherence based on timing of treatment.	Patients who met all three criteria (amount of medication consumed, type of medication and correct time of consumption) were considered adherent; those who failed any criterion were considered non‐adherent.	6/8
Thiam (2007) [25]	Senegal	Control: 744 Intervention: 778	ECRG	Active outreach to promote TB treatment adherence via DHC teams, patients and community members. Health professionals received training. During the intensive phase, medication was retrieved weekly from a health post by the DOT provider and given every 15 days. Patients at risk of abandonment were visited and encouraged to continue treatment. **Classification**: Community education **Duration**: 10 months	Usual care at NTCs (diagnosis and treatment of TB, follow‐up by TB control unit). **Duration**: 10 months	Primary: TB cure (success). Secondary: Non‐adherence to treatment (defaulted).	A cured patient was one who had a negative sputum smear in the last month and on at least one prior occasion. Those who completed treatment without negative tests were considered treatment‐completers. The number of adherent patients was calculated by subtracting the number of patients who defaulted from the total number of included patients.	9/13
Liu et al. (2015) [32]	China	Control: 1.104 Intervention: 3.069	Cluster‐randomised trial	Three strategies: text message reminders, medication monitoring or a combination. Classification: SMS Classification: Electronic Devices Classification: Electronic Devices + SMS **Duration:** 6 months	Self‐administered treatment, DOT supervised by family members, or DOT supervised by healthcare professionals. **Duration:** 6 months	**Primary:** Treatment adherence. **Secondary:** Treatment abandonment rate, sputum conversion at 2 months.	Adherence to treatment was defined as the percentage of months in which at least 20% of doses (equivalent to missing three out of 15 doses) were missed (“poor adherence”). Other adherence parameters were considered for secondary outcomes.	9/10
Wu et al. (2023) [33]	China	Control: 88 App: 82 Smart Pillbox: 90	Cohort	Sistema mHealth com lembretes via app ou smart pillbox. **Classificação**: SMS **Classificação**: Eletronic Devices **Duração**: 6–12 meses.	Self‐administered treatment, DOT supervised by family or community workers. **Duration**: 6 months	**Primary**: Treatment adherence. **Secondary**: Dropout rate, sputum conversion in 2 months. Tertiary: Treatment success.	Treatment adherence was defined as having < 20% of months with missed doses (≈15 doses). Patients with ≥ 20% missed doses were considered non‐adherent. Treatment success was defined as the sum of cured and treatment completed	8/10
Velen et al. (2023) [34]	Vietnam	Control: 126 Intervention: 124	ECR	Participants received the MERM device. The intervention consisted of two components: (1) a daily audible alert reminding the patient to take the medication; (2) feedback to health professionals about adherence data from the previous month. Patients were invited to a 15–30 min counselling session during each follow‐up visit. **Classification**: Electronic Devices **Duration**: 4 months	Participants received medications in a MERM device with the alert function disabled, used only to track openings. No feedback on adherence was given to health professionals. **Duration**: 4 months	**Primary:** Low treatment adherence. **Secondary:** Proportion of treatment months with at least 14/30 missed doses, and proportion of total expected doses missed during treatment. Tertiary: Treatment success.	Low adherence was defined as the proportion of treatment months in which ≥ 20% of doses were missed (equivalent to missing ≥ 6/30 doses) Defined as the sum of TB patients who were cured and those who completed treatment.	11/13

Abbreviations: CBIA, Community‐Based Interactive Approach; cRCT, Cluster Randomised Controlled Trial; DHC, Health Centres; DOT, Directly Observed Therapy; DOT, Directly Observed Treatment; MERM, Medication Event Reminder Monitor; NTCP, National TB Control Program; OBS‐CS, Observational – Cross‐Sectional; QES, Quasi‐Experimental Study; RCT, Randomised Controlled Trial; SMS, Short Message Service; TB, Tuberculosis; WOT, Wirelessly Observed Therapy.

### 3.3 Risk of Bias in Studies

**FIGURE 2:**
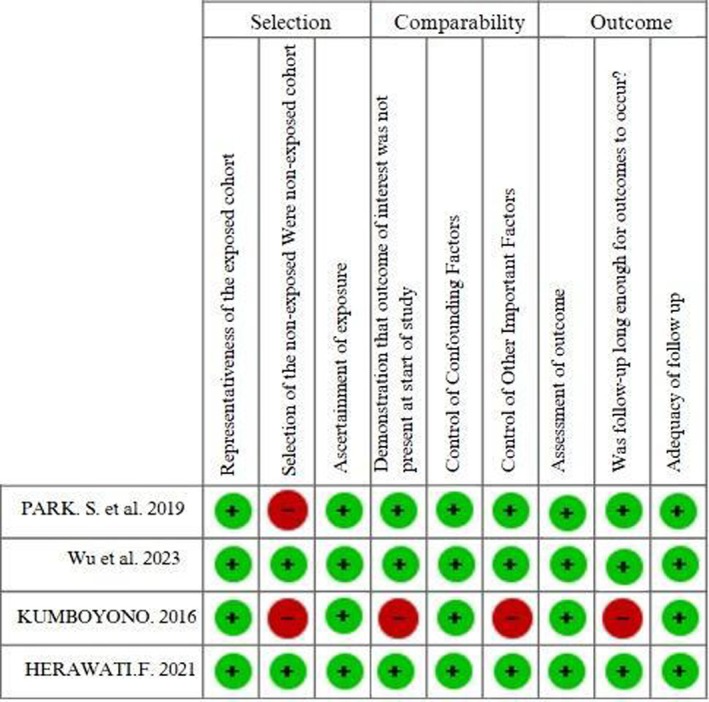
Risk of bias assessment using the Newcastle‐Ottawa scale.

**FIGURE 3:**
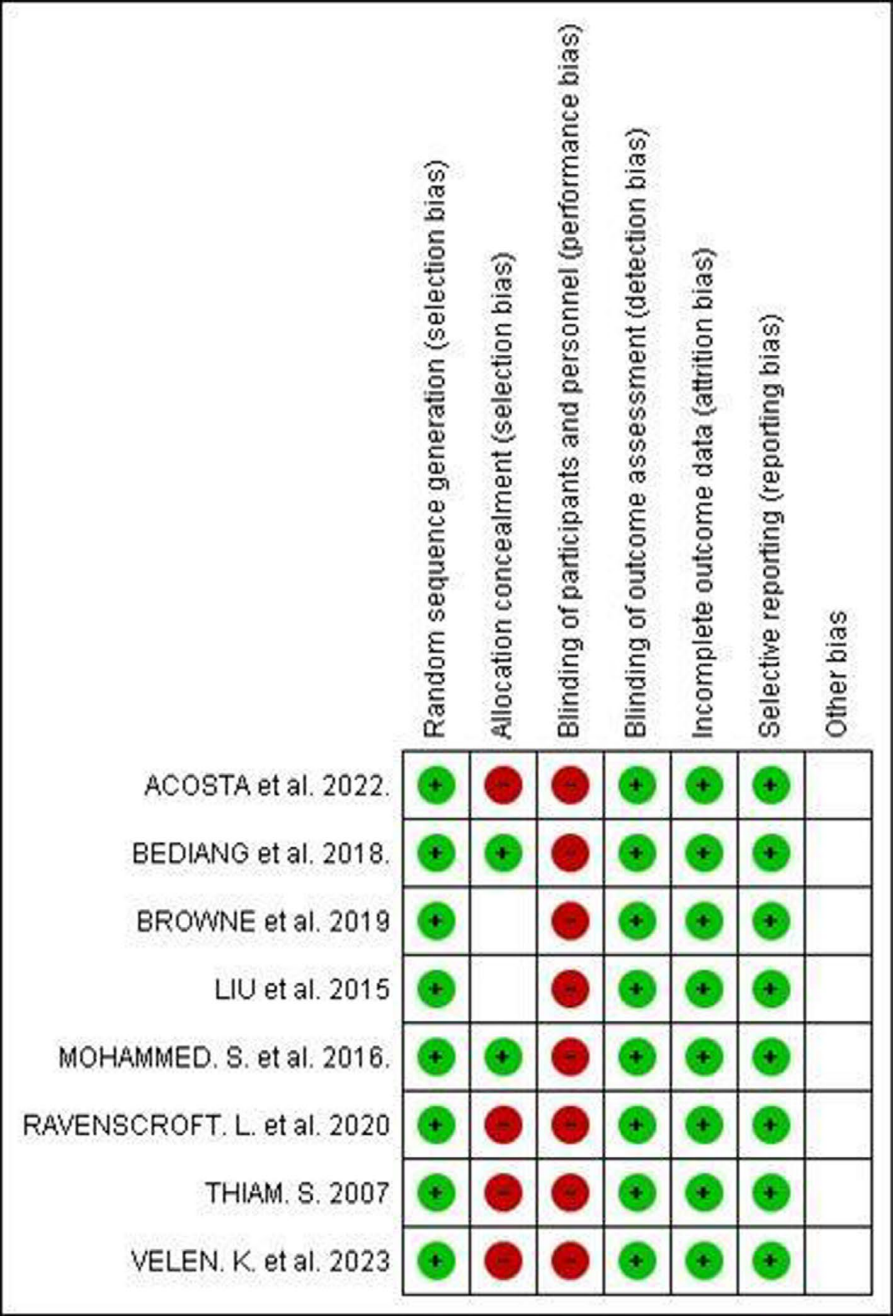
Risk of bias assessment using RoB 2.

The risk of bias was assessed using RoB 2 for randomised clinical trials, and the Newcastle‐Ottawa Scale for cohort, longitudinal and quasi‐experimental studies. The results are visualised in Figures 2 and 3.

The communication strategies were implemented both independently and in combination, and were classified into five main categories: (I) electronic devices for medication storage combined with reminder and monitoring systems [23, 28, 30]; (II) health education using community language and/or educational videos [25, 26]; (III) sending short messages via smartphones (SMS) [29, 30, 31, 32, 33]; (IV) digital services, such as electronic medication monitoring [32, 33, 34]; and (V) video observed therapy (VDOT) [24, 27].

In all studies included in this systematic review and meta‐analysis, participants in the control group received the usual treatment for tuberculosis, which consisted of standard therapy or directly observed treatment (DOT).

### 3.4 Meta‐Analysis

A total of 12 studies were included in the meta‐analysis related to tuberculosis treatment adherence. The methods used to measure adherence varied between studies, and to ensure fidelity to the original methodological approaches, the adherence criteria described by each individual study were adopted. The specific parameters used to classify participants as adherent or non‐adherent are presented in Table 1.

The combined analysis showed a pooled relative risk of 0.60 (0.47 to 0.78) for tuberculosis treatment adherence with the use of communication strategies, according to the random effects model.

**FIGURE 4:**
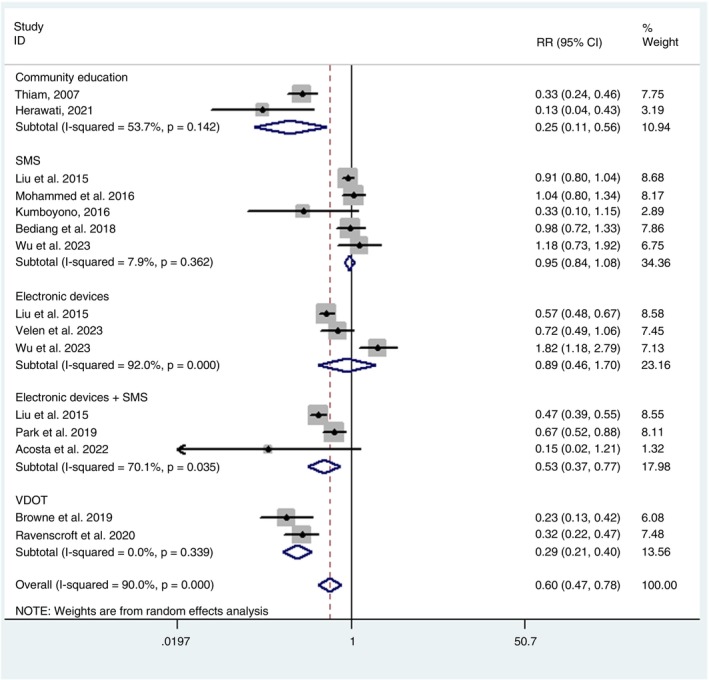
Forest plot of tuberculosis treatment adherence.

As illustrated in Figure 4, the methods that showed the greatest positive impact on adherence were community education, with a relative risk of 0.25 (0.11 to 0.56) and video observed therapy (VDOT), with 0.29 (0.21 to 0.40). With intermediate values, there were the combined use of electronic devices and sending SMS messages, which were also effective, with a relative risk of 0.53 (0.37 to 0.77). On the other hand, the methods that did not show statistical significance were the use of electronic devices alone, with a relative risk of 0.89 (0.46 to 1.70) and sending SMS alone, with 0.95 (0.84 to 1.08).

Regarding therapeutic success, eight studies were included in the respective meta‐analysis. Similarly to the adherence analysis, the definition of “success” was adopted according to the specific criteria established by each study, as detailed in Table 1. The pooled relative risk for treatment success with the use of communication strategies was 0.69 (0.49 to 0.98), also estimated using a random effects model.

**FIGURE 5:**
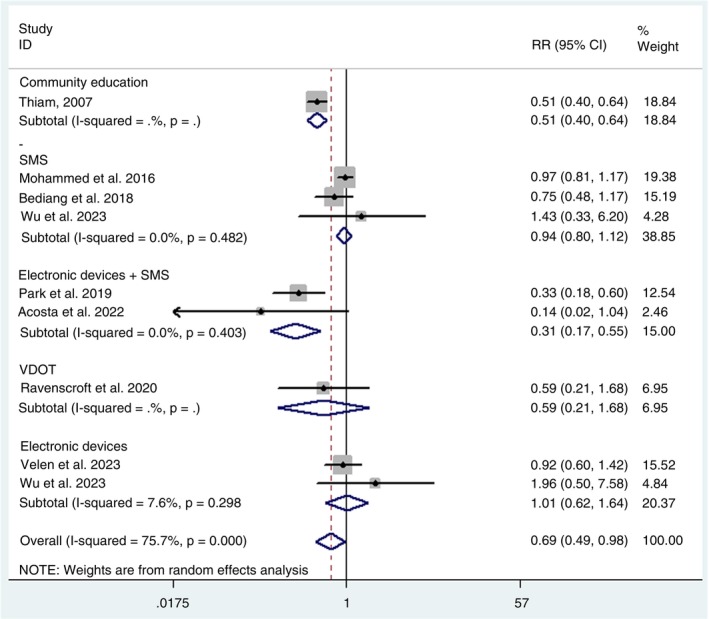
Forest plot of tuberculosis treatment success.

In Figure 5, it can be observed that the methods with the greatest efficacy for treatment success were the combined use of electronic devices and sending SMS messages, with a relative risk of 0.31 (0.17 to 0.55) and community education, with 0.51 (0.40 to 0.64). The other three interventions did not show statistical significance, namely: VDOT with a relative risk of 0.59 (0.21 to 1.68), sending SMS alone with 0.94 (0.80 to 1.12) and the exclusive use of electronic services, with 1.01 (0.62 to 1.64).

The studies included in both meta‐analyses were assessed for heterogeneity and publication bias.

**FIGURE 6:**
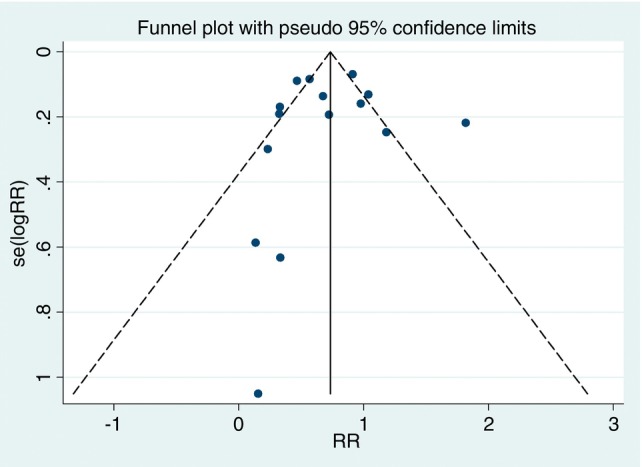
Funnel plot of adherence.

**FIGURE 7:**
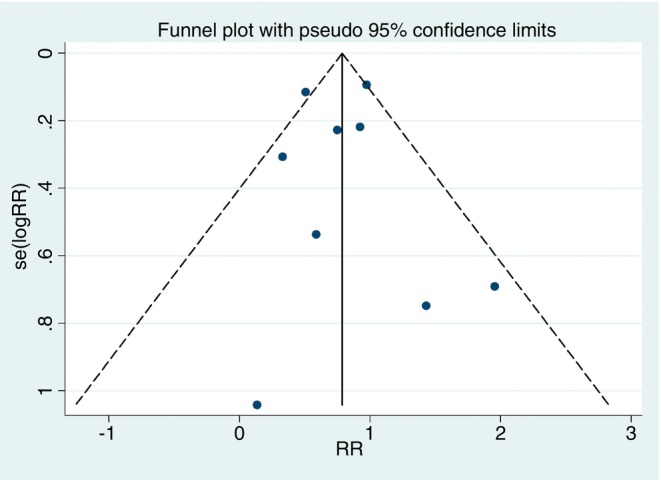
Funnel plot of success.

The publication bias analysis for adherence using the Egger test did not indicate statistical significance (*p* = 0.892), suggesting the absence of bias. This interpretation was reinforced by visual inspection of the funnel plot (Figure 6). The Egger test for treatment success also did not indicate a publication bias risk (*p* = 0.969), a result that was corroborated by the visual analysis of the funnel plot (Figure 7).

In the meta‐analysis related to adherence, a borderline difference was identified between the SMS‐based methods and VDOT (*p* = 0.055), suggesting a possible trend toward the superiority of one method over the other, although without robust statistical significance. On the other hand, in the meta‐analysis evaluating intervention success, a statistically significant difference was observed between community education and SMS use, with *p* = 0.038, indicating a more favourable effect of one approach over the other.

Significant heterogeneity was observed among the studies in the adherence to treatment meta‐analysis (*I* ^2^ = 90%; *p* < 0.001) (Figure 4). Regarding the therapeutic success meta‐analysis, significant heterogeneity was also identified (*I* ^2^ = 75.7%; *p* < 0.001) (Figure 5).

To assess the potential impact of conceptual heterogeneity arising from the different definitions of treatment adherence and success, we conducted univariable meta‐regression analyses in both meta‐analyses (adherence and success). The various definitions adopted by the studies were used as independent variables. The results indicated that the definitions used for adherence and treatment success did not significantly influence the observed effects of the communication strategies. None of the models showed a statistically significant association between outcome definitions and the magnitude of the effects. In contrast, the type of intervention showed a statistically significant association with the variation in outcomes (Figures 4 and 5).

## 4 Discussion

The settings in which the interventions included in this systematic review were conducted—with the exception of the United States of America, Senegal and Morocco—belong to at least one of the three global lists of high tuberculosis burden countries established by the WHO for the period 2021 to 2025 [4]. This indicates that most of the studies were carried out in contexts with a high incidence of the disease, which strengthens the relevance and applicability of the findings.

Use of electronic devices was observed in 10 of the 12 studies analysed in this systematic review, with only two interventions not making use of such tools. Digital technologies are mentioned as part of the third pillar of the “End TB Strategy” [4], from the Global Plan to End TB 2023–2030 [35]. Among other goals, these approaches aim to strengthen treatment adherence through the development of new tools and tactics for managing the disease.

Regarding the impact of communication strategies, the isolated use of SMS reminders did not show a statistically significant effect on treatment adherence [29, 30, 31, 32, 33] or success [29, 30, 33]. These findings contrast with evidence supporting the use of SMS to improve adherence and treatment outcomes in other chronic conditions, such as HIV (Human Immunodeficiency Virus), diabetes and asthma [36]. It is concluded, therefore, that not all SMS interventions are effective. Studies suggest that less frequent text message interventions, with content and delivery intervals individually adapted, may have a more significant effect [37]. In this sense, the lack of personalised engagement and the excessive number of messages sent to participants may have been factors that contributed to the failure to improve both adherence and treatment success.

Just as the isolated use of SMS messages, the independent use of electronic technologies also did not demonstrate statistical significance in relation to treatment adherence [32, 33, 34] or therapeutic outcomes [33, 34]. In Wu's study [33], the duration of treatment was significantly shorter in the intervention group compared to the control group, requiring a lower medication burden. However, this difference did not translate into higher adherence. On the other hand, the study by Liu [32], on its own, demonstrated greater adherence in the intervention group. This result was attributed to the daily reminder sent at the same time, which avoided the fatigue commonly associated with frequent SMS messages. The studies also emphasise the importance of the reliability of electronic technologies, which generate objective and verifiable data—something that is not always guaranteed with traditional methods used in control groups.

Conversely, interventions that combined electronic devices with SMS messaging showed greater effectiveness in both treatment adherence [23, 28, 32] and success [23, 28], with statistically significant results. The article by Acosta [23] reports that the real‐time monitored medication system helps ensure that patients are taking their medication correctly, which promotes stricter control of treatment adherence. Furthermore, frequent communication between monitors and patients allowed for early detection of issues and prompt implementation of solutions. In the study by Park [28], electronic services even helped locate patients who had abandoned treatment, enabling interventions aimed at bringing them back to therapy. Additionally, the combination of electronic systems and SMS promotes greater autonomy and engagement of the patient in their own treatment, contributing to the individual's active role in their recovery.

Regarding the studies that evaluated community education strategies [25, 26], a significant positive impact was observed on both treatment adherence [25, 26] and treatment success [25]. In Thiam's study [25], training health professionals in communication, rapport building and counselling contributed to improving relationships with patients. Similarly, in the study by Herawati [26], the use of educational videos and group discussions in the local language facilitated patients' understanding of the disease and its treatment. These results demonstrate that educational interventions tailored to the patients' cultural context and focused on the interaction between health professionals and the community can strengthen patient engagement and enhance both adherence and therapeutic success.

The VDOT method showed mixed results when comparing treatment adherence and therapeutic success. Regarding adherence, the included studies [24, 27] showed significant outcomes, which may be attributed to the reduced time patients spent travelling to clinics for DOT, as well as greater autonomy in managing their own treatment. Another important factor is the use of Wirelessly Observed Therapy (WOT) in the study by Browne [24], which employs sensors to confirm medication ingestion, making the process more accurate, effective and practical for patients. However, treatment success did not show statistically significant results. A possible explanation is that only one study assessed this outcome, limiting the sample size and, consequently, the statistical power. Moreover, factors such as drug resistance or other associated clinical conditions may contribute to this discrepancy between high adherence and failure to achieve disease cure.

Regarding the control groups in the studies analysed in this review, eight used Directly Observed Therapy (DOT) as the standard method. Three studies followed the conventional treatment provided by local clinics or hospitals. One study provided participants in the control group with an electronic device with the reminder function disabled, used exclusively to record the doses taken.

One key limitation of this study is the diversity of study designs included in the systematic review. In order to encompass the largest possible number of publications on the topic, the authors chose not to restrict inclusion exclusively to randomised controlled trials. This decision aimed to broaden the sample analysed and allow for the construction of a forest plot with greater statistical power.

Another important factor to consider refers to the different methodologies used to assess treatment success and adherence in each analysed intervention. As described in the results section, the authors adopted the outcome definitions established by each included study. Although this variation could represent a limitation, the univariable meta‐regression analyses did not indicate that this conceptual heterogeneity significantly impacted the results. Therefore, despite methodological differences, the influence of these variations on the observed statistical heterogeneity was considered minimal. Nonetheless, caution is recommended when interpreting the overall conclusions, and readers are encouraged to consult the original studies for more detailed information on the classifications used.

## 5 Conclusion

Community education strategies, VDOT and the combination of electronic devices with SMS demonstrated effectiveness in promoting adherence to tuberculosis treatment, showing statistical significance in the analyses conducted. However, when evaluating treatment success, only two interventions showed statistically significant results: community education and the combined strategy of electronic devices with SMS. Both presented positive effects on both adherence and treatment success, which suggests, based on the conducted meta‐analysis, that these are the most promising approaches for application in populations affected by tuberculosis.

Another relevant aspect of the study was the high heterogeneity observed in the meta‐analysis results for both treatment success and adherence outcomes. This variability may be related to the high effectiveness of Directly Observed Therapy (DOT) when properly implemented, which could influence the effects of the evaluated interventions. Additionally, heterogeneity may also stem from methodological differences and potential limitations in the design of the included studies.

Considering the multifactorial nature of tuberculosis and its social determinants, the interventions investigated were not able to address all the singularities and factors that influence adherence and treatment success. Nevertheless, studies that employed health education strategies and promoted active patient and community participation in the treatment—combined with the use of novel electronic technologies—showed more favourable results. These studies highlight the effectiveness of approaches that foster therapeutic bonds and stimulate patients' self‐awareness, encouraging them to recognise their capacity to change their own reality and play an active role in their treatment.

## Ethics Statement

The authors have nothing to report.

## Conflicts of Interest

The authors declare no conflicts of interest.

## Data Availability

The data that support the findings of this study are available from the corresponding author upon request.
